# High-throughput sequencing reveals hub genes for human early embryonic development arrest *in vitro* fertilization: a pilot study

**DOI:** 10.3389/fphys.2023.1279559

**Published:** 2023-11-08

**Authors:** Wuwen Zhang, Kai Li, Shifeng Li, Rong Lv, Jie Ma, Ping Yin, Li Li, Ningyu Sun, Yuanyuan Chen, Lu Lu, Yun Li, Qinhua Zhang, Hua Yan

**Affiliations:** ^1^ Reproductive Medicine Center, Shuguang Hospital Affiliated to Shanghai University of Traditional Chinese Medicine, Shanghai, China; ^2^ Center for Excellence in Molecular Cell Science, Chinese Academy of Sciences, Shanghai, China; ^3^ School of Traditional Chinese Medicine, Shanghai University of Traditional Chinese Medicine, Shanghai, China; ^4^ School of Acupuncture-Moxibustion and Tuina, Shanghai University of Traditional Chinese Medicine, Shanghai, China

**Keywords:** early embryonic development, biological information data mining, gene expression, embryonic development arrest, cell cycle, RNA-seq, ubiquitination

## Abstract

Many clinical studies have shown that embryos of *in vitro* fertilization (IVF) are often prone to developmental arrest, which leads to recurrent failure of IVF treatment. Early embryonic arrest has always been an urgent clinical problem in assisted reproduction centers. However, the molecular mechanisms underlying early embryonic development arrest remain largely unknown. The objective of this study is to investigate potential candidate hub genes and key signaling pathways involved in early stages of embryonic development. RNA-seq analysis was performed on normal and arrest embryos to study the changes of gene expression during early embryonic development. A total of 520 genes exhibiting differential expression were identified, with 174 genes being upregulated and 346 genes being downregulated. Upregulated genes show enrichment in biosynthesis, cellular proliferation and differentiation, and epigenetic regulation. While downregulated genes exhibit enrichment in transcriptional activity, epigenetic regulation, cell cycle progression, cellular proliferation and ubiquitination. The STRING (search tool for the retravel of interacting genes/proteins) database was utilized to analyze protein-protein interactions among these genes, aiming to enhance comprehension of the potential role of these differentially expressed genes (DEGs). A total of 22 hub genes (highly connected genes) were identified among the DEGs using Cytoscape software. Of these, *ERBB2* and *VEGFA* were upregulated, while the remaining 20 genes (*CCNB1, CCNA2, DICER1, NOTCH1, UBE2B, UBE2N, PRMT5, UBE2D1, MAPK3, SOX9, UBE2C, UB2D2, EGF, ACTB, UBA52, SHH, KRAS, UBE2E1, ADAM17* and *BRCA2*) were downregulated. These hub genes are associated with crucial biological processes such as ubiquitination, cellular senescence, cell proliferation and differentiation, and cell cycle. Among these hub genes, *CCNA2* and *CCNB1* may be involved in controlling cell cycle, which are critical process in early embryonic development.

## Introduction

*In vitro* fertilization (IVF) is growing rapidly, and research is focused on improving the probability of a live birth. IVF research focuses on the developmental dynamics of the preimplantation embryo, which is incubated *in vitro* for a maximum of 5–7 days. Only embryos with the highest developmental potential are selected for transfer (Sfakianoudis et al., 2021). Previous studies have shown that 10 to 15 percent of IVF embryos in humans will be permanently arrested at the 2 to 4 cells cleavage stage exhibiting no signs of apoptosis in culture, and approximately 40 percent of patients will be has at least one arrested embryo during each treatment cycle (Betts and Madan, 2008). Less than 50% of IVF embryos reach the blastocyst stage, and many do not continue to develop after transfer (Cairo Consensus Group, 2020). Several mechanisms have been proposed to explain this arrest, specifically, failure of zygotic genome activation, delayed elimination of maternal RNA, and aneuploidy et al. However, our knowledge regarding the molecular mechanisms underlying early embryonic developmental defects is still limited.

Microarrays has proven to be an efficient tool for high-throughput analysis of transcriptomes in tissues, cell lines, or embryos, particularly in the context of various stages or treatments. Some studies have utilized microarrays to investigate gene expression kinetics during early embryonic development (Huang et al., 2010). Previous studies have demonstrated that the dynamics of gene expression and defined subsets of genes regulated during preimplantation development of bovine and human embryos, particularly those related to embryonic genome activation (Misirlioglu et al., 2006; Asami et al., 2022). Epigenetic regulation has dynamic variations and may have very important functions during the process of early embryonic development (Skvortsova et al., 2018). Our previous studies have shown that *PRMT7* plays a significant role in early embryonic development (Zhang et al., 2015; Zhang et al., 2018; Zhang et al., 2021). Several studies indicated that components of the apoptotic and survival pathways were expressed during early mouse (*in vivo*) and human (*in vitro*) embryonic development using a cDNA microarray technology (Haouzi et al., 2018). However, no research has directly investigated gene expression changes associated with abnormal early embryonic development or development arrest of embryos. High-throughput sequencing (HTS) of the transcriptomes studies is a newly invented technology alternative to microarray analysis. Although it is still relatively more expensive than microarray, it presents the overall gene expression of embryo and are particularly better at detecting transcription of unknown genes at a more precise and accurate level, which can quantitatively reveal gene expression defects caused by embryonic development-associated changes in gene regulation and epigenetic variation (Wu et al., 2018). HTS has been widely used to study gene expression during the course of human early embryonic development. For example, early embryonic development *in vitro* and *in vivo* has been analyzed by HTS, and transcriptomic and epigenetic dynamics has critical roles in the epigenetic regulation of the development of mammalian embryos (Zhou et al., 2019). A recent investigation demonstrated that DEGs and miRNAs in villi from early embryonic arrest and normal pregnancy patient vis HTS, which were associated with MAPK signaling pathway, cell proliferation, and microtubule formation to impact early embryo development (Yang et al., 2019). Thus, HTS can not only help us study how genes are expressed and regulated, but also help us identify potential candidate hub genes and key signaling pathways involved in early stage of embryonic development by Bioinformatics data mining tools.

In this study, we investigated the transcriptome profile of embryos from developmental arrest and developmental normal using HTS, with the objection of identifying potential candidate hub genes and key signaling pathways which affect the early embryonic development in human. Firstly, we screened the sequencing data, enriched the function and signaling pathways of DEGs, and then constructed a protein interaction network, and finally identified the hub genes and key significant signaling pathways related to early embryonic development. Our study provides a theoretical basic for early embryonic development and may also provide insights into future strategies for preventing early embryonic arrest.

## Materials and methods

### Human embryos collection and culture

From November 2020 to November 2021, discarded embryos that were cultured *in vitro* from patients undergoing IVF-ET (*in vitro* fertilization and embryo transfer) treatment at the Reproductive Medicine Center of Shuguang Hospital Affiliated to Shanghai University of Traditional Chinese Medicine were utilized for this experiment. Counseling was offered to all individuals who donated their discarded embryos prior to signing an informed consent form.

Early development embryos were collected and cultured *in vitro* to 8∼cell stage using G-1 (Vitrolife) human embryo culture medium. Control embryos (at the 6–8 cell stage) came from polyspermic embryos and were collected on ‘day 3’ after fertilization. Embryos arrested at or before the five-cell stages were collected on ‘day 3’ after fertilization, and these arrested embryos showed no signs of degeneration on ‘day 5’ after fertilization. All embryos manipulation was approved by the Ethics Committee of Shuguang Hospital Affiliated to Shanghai University of Traditional Chinese Medicine (Approval Number: 2020-866-75-01). There are two groups. A total of six embryos were analyzed, including control groups (three polyspermic embryos) and developmental arrested groups (three discarded embryos).

### The sequencing and analysis of transcriptomic data

The analysis of SMART (Switching Mechanism at 5′ End of RNA Template)-mRNA expression involved the use of RNA sequences. The library construction process adhered to the manufacturer’s (Illumina) guidelines, utilizing high-grade RNA samples (Using Agilent 2100 Bioanalyzer to evaluate RNA integrity). The Quick-RNA Microprep Kit reagent (Zymoresearch, United States) was used to isolate total RNA from embryos. To generate both single and double-stranded cDNA, we employed the A SMARTer Ultra-low RNA sequencing kit (Takara, CA, United States) for the synthesis of cDNA from total RNA samples. 100pg-10 ng total RNA was used as starting material. PCR-amplified cDNA is purified by SPRI beads. The cDNA was validated using an Agilent 2100 Bioanalyzer and the high-sensitivity DNA Chip from an Agilent High-sensitivity DNA kit (Agilent Technologies, Santa Clara, CA). The Covaris AFA system was used to fragment the double-stranded cDNA. This was followed by purification, terminal repair, dA tail addition, joint ligation, and DNA fragment enrichment. The Agilent Technologies 2100 Bioanalyzer is used to analyze DNA size using a specific chip called the Agilent DNA-1000. The qubit User Guide was followed to effectively utilize the qubit Quantization Library (invitrogen, CA, United States). These libraries were then pooled and sequenced on Hiseq 3,000 system (Illumina) at Genergy Biotech. Co. Ltd. (Shanghai, China). The first step involved using custom perl scripts to process the raw data in fastq format (raw reads) following sequencing. In this stage, the data will be purified by removing reads containing adapters, ploy-N, and low-quality reads from the original data. Concurrently, the clean data was analyzed to ascertain the Q20, Q30, and GC content. The downstream analyses were conducted using high-quality, clean data. The reference genome and gene model annotation files were downloaded from the genome website. Reference genome indexes were constructed using Bowtie v2.2.3 and end-cleaning reads were compared with reference genomes using TopHatv 2.0.12. Gene counts were determined using HISeq v0.6.1. The FPKM (Fragments Per Kilobase of transcript per Million fragments mapped) value for each gene was calculated by dividing the number of segments by the gene’s length and the corresponding read count. FPKM is currently the most commonly used method for assessing gene expression level, taking into account the influence of sequencing depth and gene length on read count. The genes were compared using feature-counting statistics and subsequently analyzed using the DESeq2 software. Genes exhibiting a log2 fold change of ≥2 and a q value < 0.05 were categorized as differentially expressed.

### Analyze differentially expressed genes (DEGs) through the utilization of genetic ontology and pathway enrichment

We classified the DEGs into upregulated and downregulated genes for statistical analysis. The DAVID (The Database for Annotation, Visualization and Integrated Discovery) online analysis website (Version 6.8, https://david.ncifcrf.gov/tools.jsp) can be used to identify the DEGs through genetic enrichment analysis in ontology (GO). The analysis of GO enrichment included the assessment of molecular function (MF), biological process (BP), and cell component (CC). The KEGG (Kyoto Encyclopedia of Genes and Genomes) pathway analysis is conducted using the Profiler R package (Version 3.8). A significance level of less than 0.05 was considered statistically significant.

### Identify hub genes and analyze the protein-protein interaction (PPI) network

PPI was analyzed using the STRING database (version 11.0, http://string-db.org/). The protein interaction network among differentially expressed genes (DEGs) was established. To improve the reliability of the results, only protein-protein interactions with an interaction score above 0.4 were selected for constructing the PPI network. We used the Cytoscape software (Version 3.9.1, http://www.cytoscape.org/) to visualize and analyze the interaction network of DEGs based on STRING. We eliminated specific nodes and edges. The MCODE (Molecular Complex Detection) plug-in tool in Cytoscape was employed to analyze the module of the PPI network and identify the hotspot module of the DEGs network. We chose MCODE scores above 4 and a minimum of 5 nodes. The filtering criteria consist of a degree cut-off exceeding 2, a Node score cut-off exceeding 2, a K-core exceeding 2, and a maximum depth of 100. The cytoHubba extension of Cytoscape is a plugin used to identify important targets and sub-networks in complex networks. The cut-off criterion (Jian and Yang, 2020) was determined based on the top 30 genes with high degrees and a MCODE score of ≥10. Then, visualization analysis of SRTING-based central gene interaction network was conducted using Cytoscape software. Finally, DAVID analyzed the GO and KEGG pathway.

### Statistical analysis

The RNA-seq data analysis was performed using R software, as well as the DAVID, STRING, and Cytoscape software platforms. The R packages DESeq2, ggplot2, and GraphPad Prism 9.0 were utilized for data visualization. A significance level of less than 0.05 was considered statistically significant. The data analysis was conducted using IBM SPSS Statistics V.20.0 software. The *t*-test was employed to analyze continuous quantitative data. The data, displaying a normal distribution, were represented as the mean ± standard deviation.

## Results

### The expression profiles of genes in the developmental normal embryos or developmental arrest embryos

**FIGURE 1:**
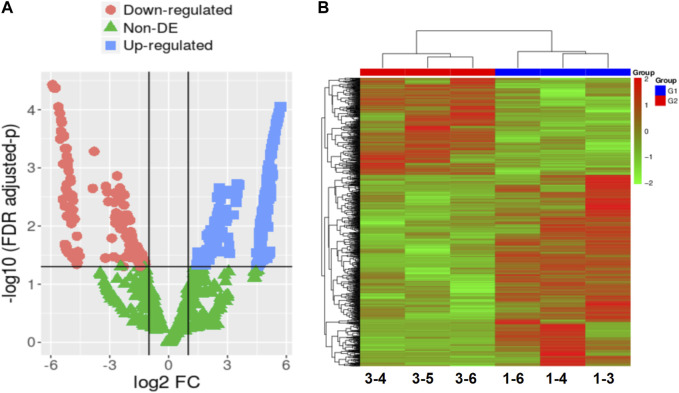
The gene expression patterns in embryos with developmental normal or embryos with developmental arrest. **(A)** In the comparison between the DNE group and the DAE group, a total of 520 differentially expressed genes (DEGs) were detected. Volcano plot is displaying DEGs between developmental normal embryos and developmental arrest embryos. The mean expression value of log10 (*p*-value adjusted) is represented on the Y-axis, while the X-axis shows the log2 fold change value. In comparison to developmental normal embryos, developmental arrest embryos are characterized by red dots indicating significantly decreased transcripts, blue dots indicating significantly increased expression (*p* < 0.05, false discovery rate (FDR) q < 0.05), and green dots representing transcripts with expression levels that did not reach statistical significance (*p* > 0.05, FDR q > 0.05). **(B)** Heatmap displaying the variation in gene expression between embryos with developmental arrest (1-3, 1-4, and 1-6) and embryos with developmental normal (3-4, 3-5, and 3-6).

RNA-seq technology was utilized to analyze gene expression changes in three developmental normal embryos (DNE) and three developmental arrest embryos (DAE) during early embryonic development. Supplementary Table S1 reveals no significant differences in age and BMI between the DNE group and the DAE group. We performed cDNA library sequencing on embryo samples obtained from the DNE and DAE groups. After filtering ribosome transcripts, we obtained a range of 50,561,686-67,400,336 paired-end reads per sample using transcriptome data software (TopHat2) and short reads map tool (bowtie). Reads that were unmapped or had multiple positions matched, accounting for 2.08%–12.36%, were excluded from further analyses. Consequently, a total of 35,498 genes were identified as expressed in the six sample, consisting of 34,208 known genes and 1,290 novel genes. After using a pairwise approach, three embryos in the same group were used to eliminate the background noise of individual-specific transcription, enabling acquisition of more relevant data from the two groups. The DESeq2 package in R was used to identify DEGs. Significantly, a total of 520 DEGs were identified in both the DNE and DAE groups (*p* < 0.01, false discovery rate (FDR) q < 0.05, and |log2FC| > 5). Among these, 346 were downregulated, and 174 were upregulated. Changes in the transcriptome can be observed using volcano plots and Heatmap, as shown in Figures 1A,B.

### Enrichment analysis

In order to assess the molecular mechanisms underlying early embryonic arrest and identify the main functional categories involved in the process, we analyzed the biological functions of the DEGs pathway using GO and KEGG on the DAVID website. Compared with the developmental normal embryos, 62 GO biological processes were identified to be under-expressed or over-expressed in developmental arrest embryos at the 0.05 significant level. Table 1 and Table 2 present the classified genes.

**TABLE 1:** Functional enrichment of downregulated genes that are differentially expressed in developmental arrest embryos *versus* developmental normal embryos.

Category	Term	*p*-value	Count
Epigenetic regulation	posttranscriptional regulation of gene expression	5.50E-29	12
	histone modification	1.20E-19	3
	protein methyltransferase activity	0.004	3
	chaperone mediated protein folding requiring cofactor	0.050	3
Ubiquitination	ubiquitin-dependent protein catabolic process	7.09E-27	13
cell cycle	regulation of mitotic cell cycle	1.45E-16	16
Cell proliferation and junction	adherens junction	4.11E-13	2
	regulation of cell proliferation	0.038	3
	cell fate commitment	0.049	2
Metabolism	cholesterol metabolic process	0.0004	7
	sphingolipid metabolic process	0.045	3
Transport	lysosomal transport	6.88E-05	5
	calcium ion transmembrane transport	0.002	7
	response to calcium ion	0.033	4
Transcription	negative regulation of transcription from RNA polymerase II promoter	0.003	22
Energy	cellular response to Camp	0.032	4
homeostasis and biosynthetic process	neuron cellular homeostasis	0.041	3
	response to nutrient	0.050	4
	ceramide biosynthetic process	0.050	3
Signaling pathway	positive regulation of MAPK cascade	0.050	3

**TABLE 2:** Functional enrichment of upregulated genes that are differentially expressed in developmental arrest embryos *versus* developmental normal embryos.

Category	Term	*p*-value	Count
cell proliferation and differentiation	cell differentiation	0.003	15
	negative regulation of T cell proliferation	0.012	3
cell adhesion	cell adhesion	0.005	10
Transcription	positive regulation of gene expression	0.006	8
	negative regulation of interferon-gamma production	0.013	2
Transport	calcium ion transmembrane transport	0.011	3
	regulation of ion transmembrane	0.026	5
development	myotome development	0.013	2
	negative regulation of neuron projection development	0.024	5
Biosynthetic process	Angiogenesis	0.006	17
	supramolecular fiber organization	0.05	2
Epigenetic regulation	positive regulation of platelet activation	0.034	2
	positive regulation of growth hormone secretion	0.037	2
	regulation of sequence-specific DNA binding transcription factor activity	0.038	2
	establishment of localization in cell	0.046	3
Metabolism	NADH metabolic process	0.05	2
Signalling pathway	adenylate cyclase-activating G-protein coupled receptor signaling pathway	0.05	6
	transmembrane receptor protein tyrosine kinase signaling pathway	0.05	5
sex differentiation	sex differentiation	0.05	2

**FIGURE 2:**
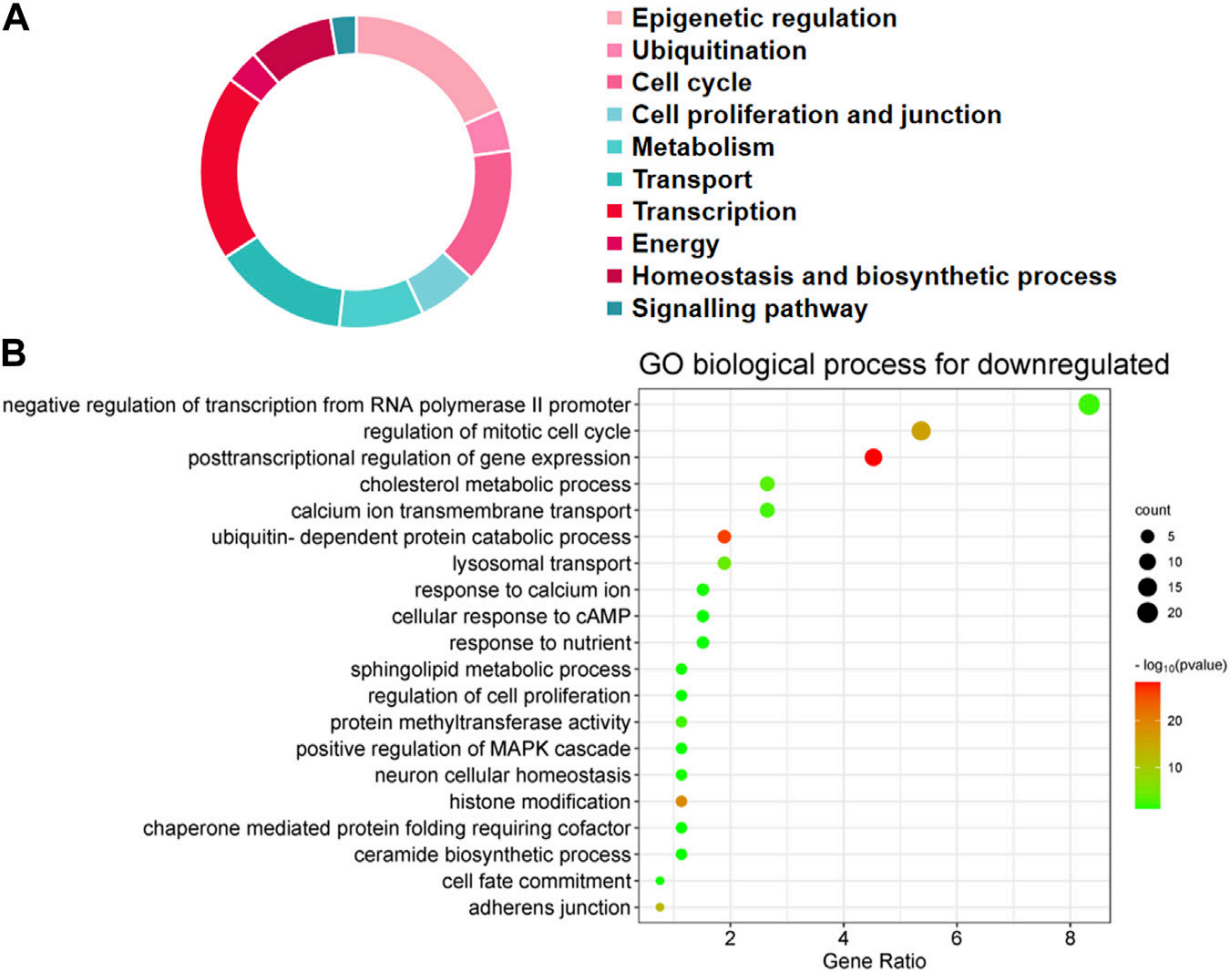
Biological processes linked to downregulated differentially expressed genes (DEGs) in embryos with developmental arrest *versus* embryos with developmental normal. **(A)** The allocation of GO annotation for biological processes of downregulated genes was divided into ten categories. **(B)** GO biological processes enrichment results for DEGs. The percentage of gene ratio is represented on the X-axis, while the Y-axis represents the GO terms. The gene count is indicated by the size of each circle. The color of circles indicates a different -log10 (P. value).

**FIGURE 3:**
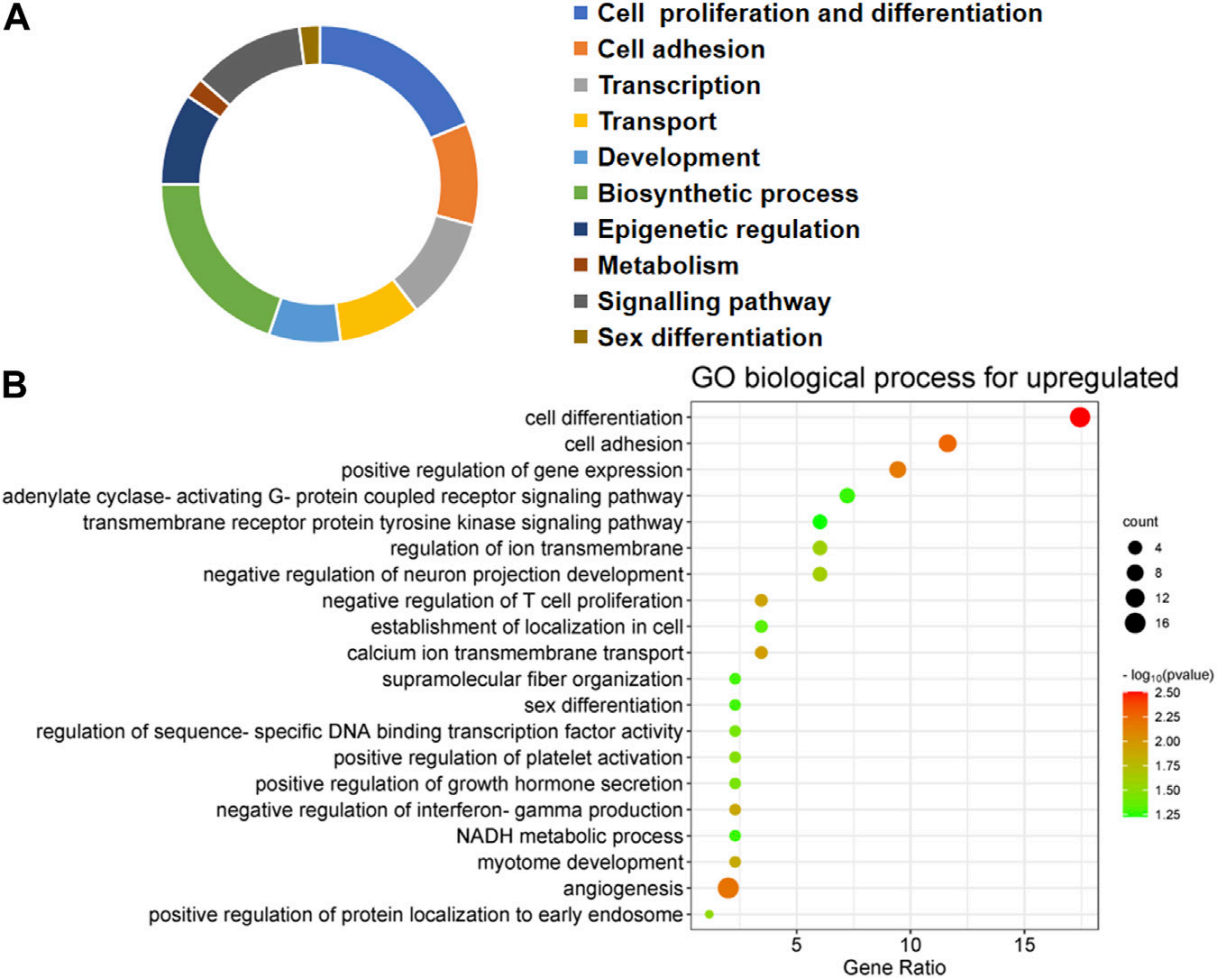
Biological processes linked to upregulated differentially expressed genes (DEGs) in embryos with developmental arrest *versus* embryos with developmental normal. **(A)** The allocation of GO annotation for biological processes of upregulated genes was divided into ten categories. **(B)** GO biological processes enrichment results for DEGs. The percentage of gene ratio is represented on the X-axis, while the Y-axis represents the GO terms. The gene count is indicated by the size of each circle. The color of circles indicates a different -log10 (P. value).

The first 20 biological processes (BP) of DEGs for downregulation are shown in Figure 2 and Table 1. These categories dominated transcription (19%), epigenetic regulation (18%), cell cycle (14%), transport (14%), cell proliferation and connectivity (6%), and ubiquitination (4%) (Figure 2A). In the transcription category, the main enrichment item was transcription from negative regulation of transcription from RNA polymerase II promoter (*p* = 0.003), which determines early embryonic development (Nguyen et al., 2022). The reduced developmental capacity of the arrested embryos may be due to the negative regulation exerted by the RNA polymerase II promoter. Moreover, epigenetic regulation terms were severely under-expressed, including posttranscriptional regulation of gene expression (*p* = 5.50E-29), histone modification (*p* = 1.20E-19), protein methyltransferase activity (*p* = 0.004) and chaperone mediated protein folding requiring cofactor (*p* = 0.05) and other cell cycle terms were also significantly reduced, for example, regulation of mitotic cell cycle (*p* = 1.45E-16) and ubiquitin-dependent protein catabolic process (*p* = 7.09E-27). However, the upregulated BP in DEGs mainly focused on biosynthesis (20%), cell proliferation and differentiation (19%), transcription (10%), epigenetic regulation (9%), and signaling pathways (Figure 3; Table 2). The category of transcription not only positively regulates gene expression (*p* = 0.006), but also negatively regulates interferon-gamma production (*p* = 0.013). The precise timing of gene expression is crucial for the development of a fertilized egg into an embryo. During embryonic development, time is usually controlled by a cascade of transcription factors (TFS). However, transcription is often inactive and the levels of transcription factors remain constant during the early stages of development, suggesting that other mechanisms are responsible for regulating the timely and organized initiation of gene expression (Pontis et al., 2022). In the epigenetic regulation category, the main enrichment terms were establishment of localization in cell (*p* = 0.046) and regulation of sequence-specific DNA binding transcription factor activity (*p* = 0.038). Signaling pathway contained the function of adenylate cyclase-activating G-protein coupled receptor signaling pathway (*p* = 0.05) and the transmembrane receptor protein tyrosine kinase signaling pathway (*p* = 0.05).

**FIGURE 4:**
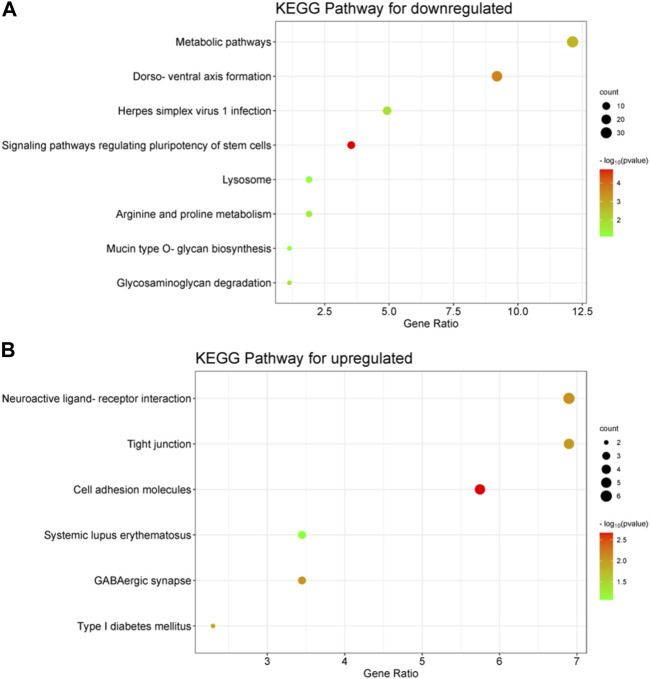
Performing KEGG analysis on differentially expressed genes (DEGs) that are downregulated **(A)** and upregulated **(B)** in embryos with developmental arrest *versus* embryos with developmental normal. *p* < 0.05 **(A)** The KEGG pathway associated with the downregulation of DEGs is mainly enriched in the formation of the dorso-ventral axis, metabolic pathways, pluripotency regulation of stem cells, and glycosaminoglycan degradation. **(B)** KEGG pathway of upregulation DEGs, which includes cell adhesion molecules, GABAergic synapse and tight junctions.

The downregulated of DEGs were predominantly enriched in binding and activity, as indicated by their molecular function (MF). Protein binding (*p* = 0.003), metal ion binding (*p* = 0.002), and transcriptional repressor activity, specifically binding to RNA polymerase II transcription regulatory region sequence (*p* = 0.002), were the main forms of ‘binding’ observed. Enriched sub-processes associated with ‘catalytic activity’ comprised histone methyltransferase activity (*p* = 0.004), ubiquitin-like protein transferase activity (*p* = 4.41E-20), glycoprotein-N-acetylgalactosamine 3-beta-galactosyltransferase activity (*p* = 0.035) and transferase activity involving glycosyl groups (*p* = 0.05) (Supplementary Figure S1A). The results of the MF analysis show that the changes in the Gene Ontology (GO) term of Biological Process (BP) are reflected in the terms ‘binding’ and ‘activity’. It was observed that the down-expressed of DEGs were predominantly localized in the cytoplasm, mitochondria, lysosomes, and membranes during the examination of cell components (CC). The CC analysis revealed that most transcripts were linked to cellular processes within the cell (Supplementary Figure S1B). The analysis of upregulated DEGs using MF also showed enrichment of various types of binding, including calcium ion binding (*p* = 0.01), binding protein binding (*p* = 0.05), and transcriptional activator activity, RNA polymerase II transcriptional regulatory region sequence-specific binding (*p* = 0.05) (Supplementary Figure S1C). The following sub-processes under ‘structural constituent’ were enriched: structural constituent of cytoskeleton (*p* = 0.05). Additionally, the CC analysis of upregulated DEGs showed major enrichment in chromatin, cell surface, adhesive junctions, anchored junctions, vesicles, and extracellular matrix, suggesting that upregulated DEGs included both intracellular and extracellular processes (Supplementary Figure S1D). The KEGG pathway associated with the downregulation of DEGs is mainly enriched in the formation of the dorso-ventral axis, metabolic pathways, pluripotency regulation of stem cells, and glycosaminoglycan degradation (Figure 4A). Figure 4B illustrates the KEGG pathway of upregulation DEGs, which includes cell adhesion molecules and tight junctions.

### Constructing PPI network and performing hot module analysis

**FIGURE 5:**
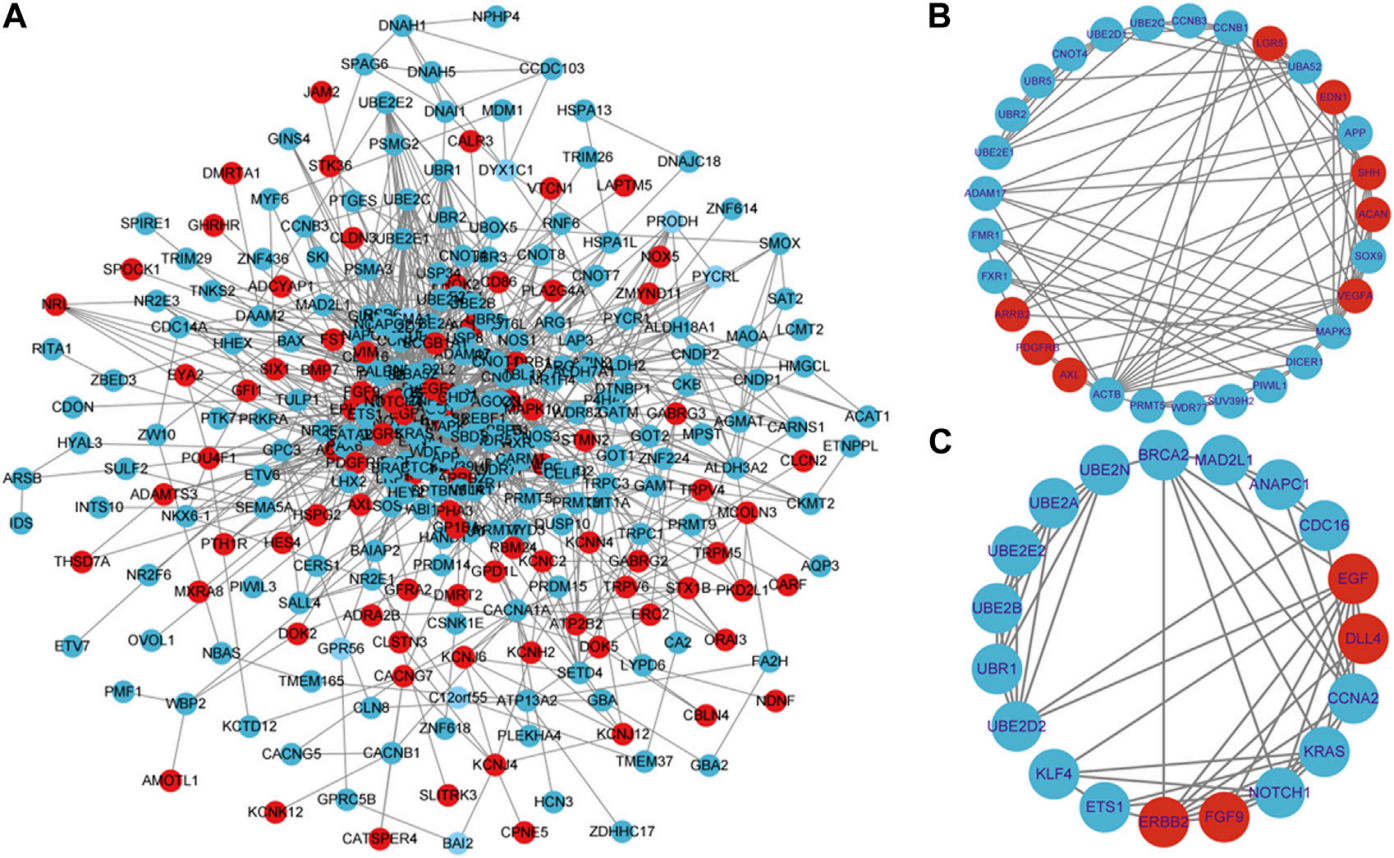
Constructing a PPI network and performing hot module analysis of differentially expressed genes (DEGs). **(A)** Cytoscape was used to build the PPI network of DEGs, which consisted of 360 nodes and 1331 edges (removing discrete nodes and edges). Genes are symbolized by circles, while protein interactions between genes are represented by lines. DEGs that were upregulated were indicated in red, while DEGs that were downregulated were indicated in blue. Protein-protein interactions with an interaction score greater than 0.4 were chosen to build a PPI network. Two significant modules were identified from the PPI network using the molecular complex detection method (MCODE) with a score of >5 and nodes >5. **(B)**: Module1 has a MCODE score of 6.37, while **(C)**: Module2 has a MCODE score of 6.0. Genes that have been upregulated are indicated in the color red, while genes that have been downregulated are indicated in the color blue.

To construct the protein-level protein-protein interaction (PPI) network of DEGs, we employed the online tool STRING. Based on the findings from the STRING database search, a network of protein interactions for DEGs has been established using Cytoscape software (Figure 5A). The entire network consists of 360 nodes and 1331 edges. The module analysis was performed using the Cytoscape plugin MCODE. MCODE has detected two modules (module 1 and module 2), with the score >5 and the node >5. Figure 5B displays Cluster 1, which comprised 29 nodes and 89 edges, with a score of 6.357. This cluster consisted of 8 upregulated genes and 21 downregulated genes. The top 10 most significant enrichment results in GO functional BP for these genes focused on protein ubiquitination, positive/negative regulation of gene expression and regulation of translation, indicating dysregulation of these biological functions in developmental arrest embryos. Cluster 2, as depicted in Figure 5C, comprises 19 nodes and 54 edges, attaining a score of 6.0. This cluster encompasses 4 upregulated genes and 15 downregulated genes. Cluster 2 genes exhibit enrichment in various processes, including ubiquitin-mediated proteolysis, cell cycle regulation, transcription and translation regulation, as well as cell migration and proliferation regulation.

### Identification of hub genes was conducted through screening

**FIGURE 6:**
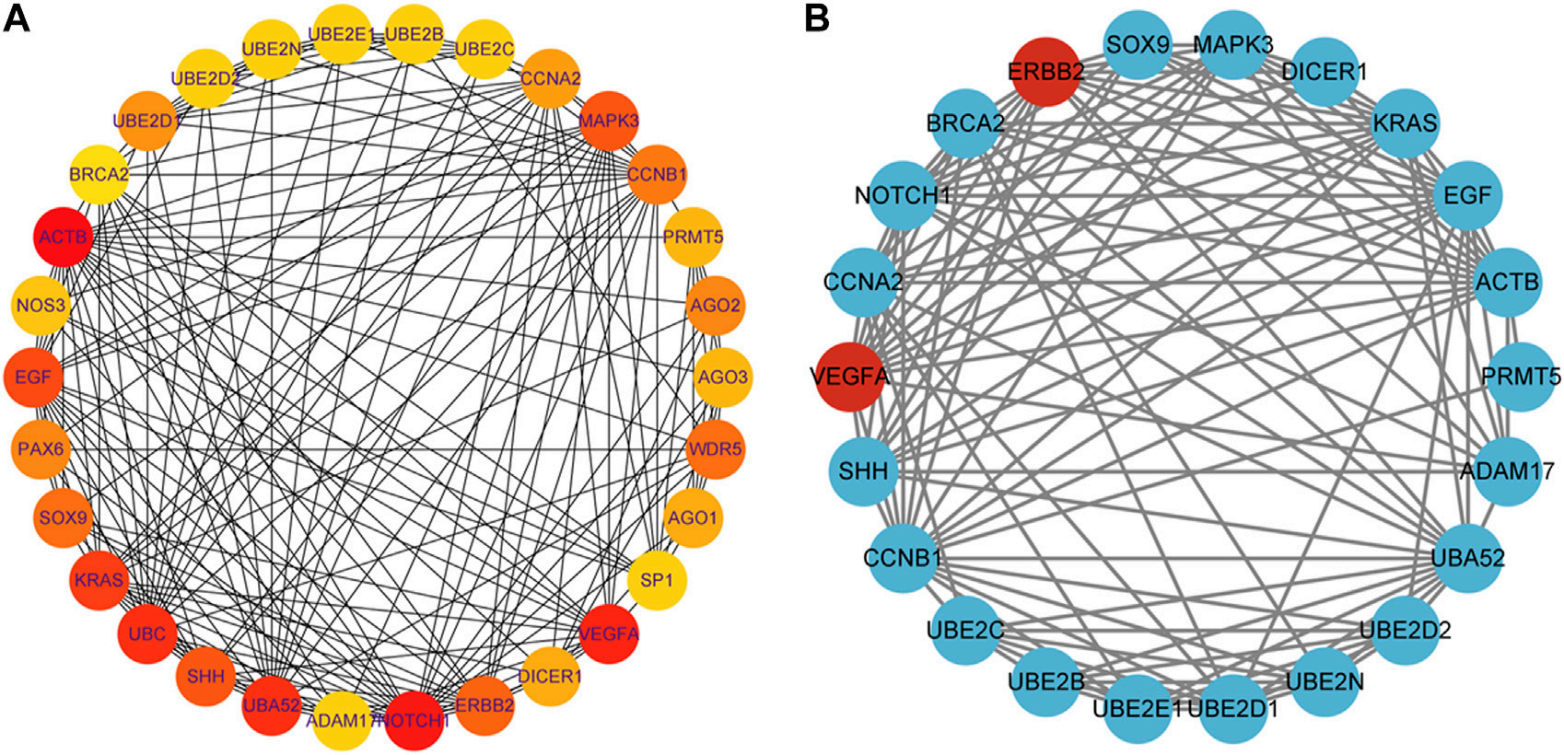
Identification of hub genes for the early embryonic development. **(A)**: The color becomes redder as the rank increases in the interrelationship network analyzed by degree in cytoHubba, with the top 30 DEGs being analyzed. **(B)**: PPI network of the hub genes. Genes that have been upregulated are indicated in the color red, while genes that have been downregulated are indicated in the color blue.

To identify hub genes associated with early human embryonic development, we utilized the STRING database in conjunction with Cytoscape software and cytoHubba plug-ins for screening. The hub genes were determined based on their high connectivity, as indicated by being among the top 30 genes (Figure 6A), and having a MCODE score of ≥6.0 (Figure 5B). Figure 6B and Table 3 show that a total of 22 hub genes were identified, with 2 showing upregulation and 20 showing downregulation in DEGs. The analysis revealed that the upregulated DEGs were *ERBB2* and *VEGFA*, whereas the downregulated DEGs included *CCNB1, CCNA2, DICER1, NOTCH1, UBE2B, UBE2N, PRMT5, UBE2D1, MAPK3, SOX9, UBE2C, UB2D2, EGF, ACTB, UBA52, SHH, KRAS, UBE2E1, ADAM17*, and *BRCA2*. The GO analysis results revealed that hub genes were predominantly enriched for the following biological processes: ‘ubiquitination’, ‘DNA transcription and expression’, ‘cell proliferation and differentiation’, etc. These abnormal signals may cause cell cycle arrest through ubiquitination and DNA transcription and expression, thus leading to the arrest of embryonic development (Table 4). Additionally, the top 10 molecular functions associated with hub genes were mainly related to ubiquitination, protein binding, and cyclin-dependent protein serine/threonine kinase regulatory activities (Table 5). DAVID conducted KEGG pathway analysis on the hub gene, identifying 10 significantly enriched pathways including cellular senescence, MAPK and *VEGF* signaling pathways, ubiquitin-mediated proteolysis, and adhesion and gap junction (Table 6). The results demonstrated that the degree and fold of *CCNA2* and *CCNB1* are dominant. Therefore, our study indicates that *CCNA2* and *CCNB1* may play an important role in the PPI network of hub genes.

**TABLE 3:** Identification of hub genes by degree and MCODE score of the Cytoscape software.

Cytoscape-MCODE				Cytoscape-cytoHubba		cytoHubba & MCODE
Name	Score	Name	Score	Name	Score	Name
SUV39H2	6.357	KLF4	6	ACTB	61	CCNB1
UBE2D1	6.357	ERBB2	6	NOTCH1	48	CCNA2
EDN1	6.357	FGF9	6	VEGFA	47	DICER1
FXR1	6.357	CCNA2	6	UBC	42	ERBB2
UBE2C	6.357	NOTCH1	6	UBA52	42	NOTCH1
ACTB	6.357	UBE2B	6	KRAS	41	UBE2B
PDGFRB	6.357	MAD2L1	6	EGF	40	UBE2N
DICER1	6.357	UBE2N	6	MAPK3	39	PRMT5
ACAN	6.357	UBR1	6	SHH	39	UBE2D1
LGR5	6.357	CDC16	6	ERBB2	35	MAPK3
SOX9	6.357	UBE2D2	6	SOX9	29	SOX9
SHH	6.357	ANAPC1	6	WDR5	29	VEGFA
UBR5	6.357	ETS1	6	CCNB1	28	UBE2C
APP	6.357	UBE2E2	6	PAX6	27	UBE2D2
UBA52	6.357	UBE2A	6	AGO2	27	EGF
AXL	6.357	DLL4	6	UBE2D1	26	ACTB
VEGFA	6.357	KRAS	6	CCNA2	24	UBA52
ADAM17	6.357	BRCA2	6	DICER1	23	SHH
PRMT5	6.357	EGF	6	AGO1	23	KRAS
ARRB2	6.357			PRMT5	22	UBE2E1
CNOT4	6.357			AGO3	22	ADAM17
UBR2	6.357			NOS3	21	BRCA2
WDR77	6.357			UBE2E1	19	
FMR1	6.357			UBE2C	19	
PIWIL1	6.357			UBE2N	19	
UBE2E1	6.357			UBE2D2	19	
MAPK3	6.357			UBE2B	19	
CCNB3	6.357			ADAM17	19	
CCNB1	6.357			SP1	19	
				BRCA2	18	

**TABLE 4:** The top 10 biological process associated with the hub genes.

Term	*p*-value	Count	Genes
positive regulation of gene expression	3.56E-07	8	SHH, NOTCH1, EGF, ERBB2, KRAS, SOX9, MAPK3, VEGFA
protein K48-linked ubiquitination	4.10E-07	5	UBE2B, UBE2C, UBE2D2, UBE2E1, UBE2D1
positive regulation of cell proliferation	5.48E-07	9	SHH, NOTCH1, EGF, ERBB2, KRAS, SOX9, ACTB, VEGFA, ADAM17
protein polyubiquitination	5.72E-07	6	UBE2B, UBE2C, UBE2D2, UBE2N, UBE2E1, UBE2D1
ubiquitin-dependent protein catabolic process	6.31E-06	6	UBE2B, UBE2C, UBE2D2, UBE2N, UBE2E1, UBE2D1
negative regulation of gene expression	9.83E-06	6	SHH, CCNB1, NOTCH1, SOX9, DICER1, VEGFA
negative regulation of cell differentiation	3.09E-05	4	PRMT5, SHH, KRAS, ACTB
positive regulation of protein phosphorylation	4.64E-05	5	ERBB2, KRAS, SOX9, MAPK3, VEGFA
cell fate specification	5.35E-04	3	SHH, NOTCH1, SOX9
positive regulation of transcription, DNA-templated	5.52E-04	6	CCNA2, SHH, ACTB NOTCH1, EGF, SOX9

**TABLE 5:** The top 10 molecular function associated with the hub genes.

Term	*p*-value	Count	Genes
ubiquitin conjugating enzyme activity	2.16E-10	6	UBE2B, UBE2C, UBE2D2, UBE2N, UBE2E1, UBE2D1
transferase activity	1.21E-08	7	UBE2B, UBE2C, UBE2D2, UBE2N, UBE2E1, UBE2D1, KRAS
ubiquitin-protein transferase activity	4.15E-06	6	UBE2B, UBE2C, UBE2D2, UBE2N, UBE2E1, UBE2D1
protein binding	4.32E-04	20	PRMT5, NOTCH1, UBE2B, UBE2C, EGF, UBE2D2, UBE2E1, UBE2D1, DICER1, ACTB, VEGFA, CCNA2, SHH, CCNB1, ERBB2, UBE2N, KRAS, SOX9, UBA52, MAPK3
ubiquitin protein ligase binding	0.003375	4	UBE2B, UBE2N, UBE2D1, UBA52
identical protein binding	0.005353	7	PRMT5, NOTCH1, ERBB2, KRAS, ACTB, MAPK3, VEGFA
ubiquitin-like protein ligase binding	0.023903	2	CCNB1, UBE2C
cyclin-dependent protein serine/threonine kinase regulator activity	0.033702	2	CCNA2, CCNB1
ubiquitin protein ligase activity	0.042871	3	UBE2C, UBE2D2, UBE2D1
receptor agonist activity	0.055888	2	EGF, VEGFA

**TABLE 6:** The top 10 KEGG pathway for the hub genes.

Term	*p*-value	Count	Genes
Ubiquitin mediated proteolysis	3.89E-07	7	UBE2B, UBE2C, UBE2D2, UBE2N, UBE2E1, UBE2D1, UBA52
Progesterone-mediated oocyte maturation	0.001348	4	CCNA2, CCNB, KRAS, MAPK3
MAPK signaling pathway	0.00338	5	EGF, ERBB2, KRAS, MAPK3, VEGFA
Cellular senescence	0.004523	4	CCNA2, CCNB1, KRAS, MAPK3
VEGF signaling pathway	0.007293	3	KRAS, MAPK3, VEGFA
Adherens junction	0.010428	3	ERBB2, ACTB, MAPK3
Regulation of actin cytoskeleton	0.011394	4	EGF, KRAS, ACTB, MAPK3
Ras signaling pathway	0.014116	4	EGF, KRAS, MAPK3, VEGFA
Gap junction	0.015713	3	EGF, KRAS, MAPK3
Phospholipase D signaling pathway	0.04131	3	EGF, KRAS, MAPK3

## Discussion

Successful human reproduction requires gamete maturation, fertilization, and early embryonic development. However, early embryonic development is a multifactorial phenomenon subject to intrinsic and extrinsic factors, and is regulated by maternal mRNAs and proteins that are loaded into the egg during oogenesis, and abnormalities in this process will lead to embryo developmental arrest and recurrent failure of IVF/ICSI treatment (Yatsenko and Rajkovic, 2019). In this study, 520 DEGs were screened by the HTS data analysis, including 174 upregulated genes and 346 downregulated genes. In addition, the analysis of DEGs using Gene GO reveals their involvement in various biological processes, molecular functions, cellular components, and KEGG pathway indicates that many of these genes seem to play crucial roles in the process of early embryonic development.

Previous study had determined the whole genome transcriptome profile and the miRNA profile of villi tissues from the early embryonic arrest patients and normal pregnancy using HTS techniques (Yang et al., 2019). The results of this study have shown that these DEGs and miRNAs are mainly enriched in cell activity (including proliferation, differentiation, migration and invasion), complement and coagulation cascades, and dilated cardiomyopathy. Hsa-miRNA 518, novel-m0045-5p and their target gene *EGR1/RMDN3* were selected as key candidate genes and miRNAs, which was involved in the development of early embryonic arrest in human. However, there are many molecular mechanisms involved in the process of early embryonic development, and it is important to explore the molecular mechanisms of early embryonic development from different perspectives for studying the efficiency of IVF treatment in embryonic development arrest. Early embryonic arrest is one of the main causes of female infertility, with genetic factors acting as a major contributing factor. To date, the etiology of early embryonic arrest remains unclear and the treatment methods are limited, making the etiology of early embryonic arrest a critical research topic.

In contrast to previous studies, the enrichment analysis of DEGs in this study mainly focused on the functions of transcription, epigenetic regulation, cell cycle, cell proliferation and connectivity, ubiquitination, and embryonic development. Molecular functions such as protein and RNA binding, transcription repressor activity, histone methyltransferase activity and ubiquitin-like protein transferase activity are highly enriched, which is consistent with the cell cycle and early embryonic development. The PPI network analysis revealed that Cluster 1 was significantly enriched in biological processes such as protein ubiquitination, regulation of gene expression (both positive and negative), and translation regulation. Cluster 2 exhibits enrichments in ubiquitin-mediated proteolysis, cell cycle regulation, transcription and translation regulation, as well as cell migration and proliferation regulation. Subsequently, 22 hub genes were screened including 2 upregulated DEGs and 20 downregulated DEGs (Figure 6B; Table 3), and among which *CCNA2* (cyclin A2) and *CCNB1* were found to be the top degree genes. The top 10 biological processes for hub genes were mainly enriched in ubiquitination, DNA transcription and expression, cell proliferation, and differentiation (Table 4), and the top 10 molecular functions for hub genes was predominantly enriched in ubiquitination, protein binding, and cyclin-dependent protein serine/threonine kinase regulator activity (Table 5). The KEGG pathway of hub genes was mainly involved in ubiquitin-mediated proteolysis, cellular senescence, MAPK signaling pathways, actin cytoskeleton regulation, and progesterone-mediated oocyte maturation and adherens and gap junction (Table 6). The top enriched GO and KEGG terms of hub genes is ‘Ubiquitination-related functions’, ‘Cellular senescence’, and ‘Cell cycle’. Therefore, we believe that the normal expression of genes in the embryos affects the process of early embryonic development. In conclusion, this study demonstrated that the molecular mechanisms of early development can be elucidated by identifying the roles of genes enriched in ‘ubiquitination’, ‘cell senescence’, and ‘cell cycle’ in early embryonic development arrest.

### Proteins ubiquitination regulates early embryonic development

Early mammalian embryos development is a unique biological process regulated by various post-translational modifications. Early embryonic development is largely controlled by specific ubiquitin-proteasome system (UPS), which is also reported to be associated with oocyte maturation and mammalian fertilization (Wu et al., 2021). In the early stages of embryonic development, the zygote genome is transcriptionally silent, and the development of the early embryo primarily depends on the maternal mRNA and proteins in the oocyte. Thus, protein-level regulation, especially ubiquitination and ubiquitin-mediated proteolysis, is essential for early embryonic development (Huo et al., 2004a; Osman et al., 2018; Sha et al., 2020). Previous studies demonstrated that over 63,000 unique ubiquitination sites on 9,200 proteins in human cell lines using mass spectrometry. They are associated with regulation cell cycle, fertilization, oocyte maturation, and early embryonic development (Akimov et al., 2018). Ubiquitination and ubiquitination-related pathway including mitotic cell cycle, and meiosis are most differentially enriched biological processes between developmental arrest and developmental normal embryos in our study. Our study indicated that the genes involved in ubiquitin-dependent protein catabolism process were downregulated in developmental arrest embryos. In addition, the results of the GO and KEGG pathway analysis reveal that ubiquitination-related items included 13 downregulated genes (*UBE2B, UBE2A, UBE2C, UBE2D2, MAD2L1, MAD2L2, UBE2N, UBE2E1, UBE2D1, CCNB1, UBA52, CDC16, UBC*) (Table 2).

Ubiquitination regulates various reproductive processes mainly by modulating protein stability. Aberrant function or regulation of ubiquitination is the root cause of developmental abnormalities (Huo et al., 2004a). Downregulated expression of hub genes such as *UBE2B, UBE2C, UBE2D2, UBE2N, UBE2E1, UBE2D1* and *UBA52* are mainly enriched in the ‘ubiquitin-mediated proteolysis’ pathway (Table 6). *UBE2C* (Ubiquitin coupling enzyme E2C), a constituent of the anaphase promoting complex/cyclosome (APC/C), is an E2 enzyme that is highly involved in the PPI network of the hub gene and promotes the degradation of diverse target proteins during the metaphase-to-anaphase transition in the cell cycle. The *UBE2C* gene controls the G2/M checkpoint and maintains genetic stability by regulating the degradation of securin (Hao et al., 2012). Our previous studies have shown that dysregulation of *UBE2C* may disrupt the meiosis process in aged oocytes (Yuan et al., 2021). Our results demonstrated that *UBE2C* downregulated in the developmental arrest embryos may be led to a decrease in cell proliferation rates and alter cell cycle profiles. *UBE2E1* is a novel member of Polycomb inhibitory Complex 1, an E3 ligase complex responsible for ubiquitinating histone H2A and repressing gene expression. Histone H2A ubiquitination plays a critical role in transcription inhibition and DNA damage response (Wheaton et al., 2017).

APC/C is an essential E3 ubiquitin ligase in eukaryotic cell division. The APC/C plays a crucial role in mitosis by promoting the degradation of key mitotic regulators, such as securin and cyclin B1 (*CCNB1*), through ubiquitylation. These regulators are required for metaphase-to-anaphase transition and cell division. Two E2 ubiquitin-conjugating enzymes, *UBE2C (UBCH10)* and *UBE2S*, are employed by the APC/C (Sivakumar and Gorbsky, 2015). *UBE2C* and *CCNB1* are the hub genes of the downregulated genes in developmental arrest embryos in our study. APC/C activation in mitosis is tightly regulated by spindle assembly checkpoint (SAC). The SAC senses kinetochores that are not attached to microtubules and generates the mitotic checkpoint complex (MCC) (London and Biggins, 2014). However, the activation of APC/C is inhibited by MCC until all chromosomes are aligned on the metaphase plate. The unattached centromere then binds the monopolar spindle 1 (*MPS1*) kinase, which activates SAC signaling (Hiruma et al., 2015). Activation of SAC induces a structural change in mitotic arrest-deficient 2 (*MAD2* or *MAD2L1*), which is essential for the formation of MCC. *MAD2L1* is involved in downregulated genes in developmental arrest embryos. Inactivated SAC invariably leads to catastrophic aneuploid in mammalian cells, and the elimination of SAC components via genetic deletions is lethal (Buffin et al., 2007). The function of *UBE2C* and *UBE2S* in mitosis and cell viability is essential. The ubiquitin-conjugating enzyme *UBE2E3*, which is conserved among vertebrates, is essential to retinal pigment epithelial (RPE) cell proliferation. Kendra et al. found that reducing the levels of *UBE2E3* causes RPE cells to exit the cell cycle (Plafker et al., 2008). Therefore, these findings indicated that dysregulation of *UBE2C, CCNB1*, and *MAD2L1* in developmental arrest embryos may impede the mitotic progression of the cell cycle. *UBA52* (Ubiquitin A-52 residue ribosome protein fusion Product 1), is an ubiquitin-ribosome fusion gene. which is a major source of ubiquitin protein for the covalent modification of proteinaceous substrates recycled via the UPS. The cDNA microarray analysis revealed a significant six-fold increase in *UBA52* expression level in blastocyst embryos compared to metaphase II oocytes (Whitworth et al., 2005). Other studies have shown that *UBA52* gene is also abnormally expressed in embryos of aberrant rhesus monkeys with reduced developmental potential (Mtango and Latham, 2007). Kobayashi et al. demonstrated that the mutation of *UBA52* in mice led to embryonic mortality. This mutation affected the ubiquitin levels in embryos, as well as ribosome assembly, cell cycle progression, and overall protein synthesis (Kobayashi et al., 2016). Although, *UBA52*-deficient mouse fetuses died before 10.5 days of embryo, they could develop into blastocysts and implantation. Studies have shown that CRISPR/Cas9 of *UBA52* significantly decreased the formation of porcine blastocysts *in vitro*, and decreased the expression of *UBA52* resulting in developmental arrest at the 4-cell to 8-cell stage and deformation of the blastomere nucleus (Mao et al., 2018). *UBA52*-deficient cells showed no change in total ubiquitin levels. However, *UBA52*-deficient cells showed reduced protein synthesis and cell cycle arrest (Kobayashi et al., 2016). Therefore, downregulation of *UBA52* expression in developmental arrest embryos may affect ubiquitin production and early embryonic development. Altogether, changes in the expression levels of these ubiquitination-related genes suggest the impairment of the balance of the ubiquitination system during the process of early embryonic development. Dysregulation of these key genes associated with embryonic development will lead to early embryonic developmental arrest.

### Cellular senescence and cell cycle regulate early embryonic development

Early embryonic development is the fastest stage of embryonic cell proliferation and differentiation, accompanied by large-scale zygotic genes transcription (Zhang et al., 2021). The precise expression of cell cycle regulators during preimplantation development is essential for the embryo to effectively respond to DNA damage, stress, and other adverse conditions by activating survival and repair mechanisms, or apoptotic processes. The timely onset of expression of key cell cycle regulators is crucial for normal embryonic development, and abnormalities may lead to cell apoptosis and the embryo developmental arrest (Chari et al., 2019). Mammalian development involves complex processes of cell division, differentiation, programmed cell death, and cellular senescence. Cellular senescence previously believed to occur only in pathological states such as senescence and tumorigenesis, however, has recently been reported to also play a crucial role in embryonic development (Wanner et al., 2020). Our previous studies also demonstrated that the arrested embryos enter a cellular senescence-like state. This state is characterized by cell cycle arrest, a decrease in ribosomes and histones, and downregulation of MYC and p53 activity (Yang et al., 2022). Cellular senescence is a cellular process initiated by inducing a permanent cell cycle arrest, increasing cellular inflammation and preventing cell proliferation, and the cell cycle state is closely associated with cell senescence (Ogrodnik, 2021). The arrest of cell cycle is one of the most defining hallmarks of cellular senescence. In our study, the four hub genes for cell cycle and cellular senescence, *CCNA2, CCNB1, KRAS*, and *MAPK3* were downregulated, while *ERBB2* was upregulated.

The RAS family consists of *NRAS, HRAS* and *KRAS. KRAS*, a member of the ras proto-oncogene family, plays an important role in cell proliferation and differentiation. It is also the member of the RAS family to play a crucial role in early embryonic development (Alves et al., 2019). Previous studies demonstrated that *KRAS* was indispensable for development and its absence could lead to embryo lethality (Minati et al., 2022). Other research indicated that *KRAS*-deficient mouse embryos develop sever growth defects and die *in utero* around E15.5. The knockout of *KRAS* in mice results in embryo development defects and increased embryo mortality (Koera et al., 1997). All of findings suggested that *KRAS* is key regulator of early embryonic development in mice. Mitogen activated protein kinase (*MAPK*) plays an important role in intracellular actions in response to various extracellular signals. Studies shown that *MAPK3* also plays a key role in the development stage of zebrafish. Knockdown of *MAPK3* will cause death 51 h post-fertilization in zebrafish with notochord and whole body distortion (Chiou et al., 2007). The normal expression of *MAPK3/1* (*ERK1/2*) in ovarian granulosa cells plays a vital role in female fertility (Fan et al., 2009). Studies have indicated that *ERK1* and *ERK2* co-expressed in all mammalian tissues and act as key regulators of cell proliferation, differentiation, and oocyte maturation (Su et al., 2002). Mice with *ERK1*-null are viable and fertile, whereas embryos from mice with the *ERK2* mutation were lethal (Aouadi et al., 2006). *ERBB2*, along with *ERBB1*, *ERBB3*, and *ERBB4*, is a member of the epidermal growth factor receptor family and plays a critical role in tumorigenesis. Previous studies shown that deletion of *ERBB2* expression in knock-out mice severely affects early embryonic development (Britsch et al., 1998). Some studies suggested that the precise threshold levels of *ERBB2* signaling is crucial for embryonic development. Interestingly, the *ERBB2* expression level is significantly higher in animals, but 5%–10% of normal *ERBB2* levels is required for early embryonic development (Chan et al., 2004). *ERBB2* was upregulated in developmental arrest embryos. However, whether the abnormal expression level of ERBB2 will affect early embryonic development in human remain to be further verified.

Embryogenesis depends on a highly coordinated cascade of genetically encode events (Lee et al., 2014). Early embryonic development includes two main molecular events, one is maternal clearance; and the second is zygotic genome activated transcription (ZGA). Maternal mRNA clearance is an essential step in the process of early embryonic development, and the abnormal expression of these maternal embryonic development genes will lead to early embryonic developmental arrest (Huo et al., 2004a). ZGA occurs mainly in the 2∼cells stage in mice and the 4–8 cells stage in humans. Due to the failure of ZGA activation, a lot of maternal mRNAs related to cell cycle that should have been degraded was not cleared and accumulated, which ultimately leads to the occurrence of embryonic development arrest (Eckersley-Maslin et al., 2018). Ubiquitin-proteasome pathway (UPP) mediated protein degradation plays a significant role in the regulation of cell cycle progression. Critical proteins that regulate cell cycle progression, including cyclins, *CDKs*, and securing, are known to be degraded by UPPs at specific cell cycle point (Huo et al., 2004b). Currently, three major cell cycle transitions are known, namely, entering S phase, seperating sister chromatids, and exiting mitosis, all of which requires the degradation of specific proteins. Entry into mitosis is regulated by M-phase or maturation promoting factors (MPF), a kinase complex composed of a regulatory subunit cyclin B and a catalytic subunit *p34cdc2* (Brantley and Di Talia, 2021). Withdrawal from mitosis or meiosis requires the inactivation of MPF, however, this is achieved through the disruption of cyclin B by UPP. Abnormal expression of cell cycle regulators will affect the process of early embryonic development. In our study, we found that the *CCNA2* and *CCNB1* genes, which are closely related to cell cycle regulation, are downregulated in embryos with developmental arrest.

### *CCNA2* and *CCNB1* may play crucial roles in early embryonic development

Due to a lack of de novo mRNA transcription during the process of early embryonic development, the regulation of protein levels, especially the regulation of UPS, is particularly critical. The cell cycle of mammalian is regulated by a variety of mechanisms, including the activity of cyclin-dependent protein kinase complexes. *CCNA2* is a highly conserved member of the cyclin family that plays a crucial role in regulating the cell cycle. *CCNA2* promotes the transition of G1/S and G2/M by binding to and activating cyclin dependent kinase 2 (*CDK2*). Thus, the process of the cell cycle is precisely controlled by cyclins and their catalytic partners, *CDKs* (Lim and Kaldis, 2013). Cyclins and *CDK* are key regulators of the cell cycle. Mammals have more than 20 cyclins and 20 *CDKs.* A gene ablation study in mice has demonstrated that most of these factors are not essential for survival and fertility, but have been revealed their functional redundancy, whereas *CCNA2, CCNB1*, and *CDK1* are essential for early embryonic development. Cyclin/*CDK* complexes play a crucial regulatory role in mitosis and meiosis during the cell cycle (Chotiner et al., 2019). Cyclins and *CDKs* work together to regulate the progression of the G1, S, G2, and M phases of the cell cycle. In the S phase, *CCNA2/CDK2* facilitates the transition from the S phase to the G2 phase. In the late G2 phase, *CCNA2* activates *CDK1* to promote the occurrence of M phase, *CCNB1/CDK1* drives the transition from G2 phase to M phase. In addition, mouse genetic studies have revealed that *CDK1, CCNA2*, and *CCNB1* are essential for the cell cycle progression of mitosis. However, mutation of the cyclin A1(*CCNA1*) gene was viable in mice, suggesting that *CCNA1* is dispensable for early embryonic development (Liu et al., 1998). In contrast, *CCNA2* is necessary for development, and the mutant genotype is lethal. Murphy et al. confirmed that the development of blastocysts with *CCNA2*-mutations stopped development immediately after implantation, with no significant changes in size and morphology (Murphy et al., 1997). These findings suggest that *CCNA2* is an essential cyclin for mitosis during early embryonic development in mice. Thus, early embryonic development arrest may be caused by the failure of the mutant embryo to ensure adequate levels of cell proliferation during organogenesis. In fact, this stage is also a critical developmental ‘checkpoint’ for other cell cycle regulators. Embryos with mutation of ubiquitin ligase (E3 enzyme), culin-1 or -3 mutations are also arrested at this stage (Dealy et al., 1999). Our results suggest that some ubiquitin genes are under-expressed in the early developmentally arrested embryos.

B-type cyclin, as *MPF*, and *CDK1* participate in the transition from G2 phase to M phase in mitosis. In mice, there are three known type B cyclins, namely, *CCNB1, CCNB2,* and *CCNB3. CCNB1* and *CCNB2* display widespread expression in various tissues (Brandeis et al., 1998). *CCNB1* plays a specific role in microtubule binding while entering the nucleus in the late G2 phase of the cell cycle, and these characteristics that may make it critical for cell mitosis. *CCNB1*-null embryos die before embryonic day 10, suggesting that *CCNB1* is essential for early embryonic development (Brandeis et al., 1998). In addition, it has been shown that *CCNB1*-null embryos are arrested in G2 phase at the 4∼cell stage after two normal mitotic divisions in culture. These data indicate that *CCNB1* is essential for G2-M transition in the mitotic cell cycle. As with CCNA2, maternal stores of *CCNB1* are likely sufficient to drive the first two rounds of embryo division (Strauss et al., 2018). Remarkably, the *CCNB1*-null embryo exhibited arrest at the 4-cell stage, which occurred earlier than the blastocyst stage arrest observed in the *CDK*-null embryo (Diril et al., 2012). In summary, the above findings suggested that the abnormal expression of these key genes may be one of the important reasons for early embryonic development arrest, by inhibiting of cell proliferation, inducting of cell cycle arrest and cellular senescence, and promoting of apoptosis.

In conclusion, our results reveal the various DEGs and signaling pathways involved in process of early human embryonic development. In addition, the findings of this study had to be seen in light of some limitations. Firstly, the number of arrested embryos from the same patient was limited, and all of arrested embryos in this study were from different patients, without considering the differences between patients’ embryos. Secondly, the source of developmental normal embryos was limited and ethical restrictions, and the control embryos in the experiment were polyspermic embryos. Our future research will focus on identifying these hub genes and elucidating their physiological functions and mechanisms. Although the results need to be further verified by molecular, cellular and animal experiments. However, these data provide some clues for future research on the molecular mechanism of early embryonic development. Our research findings suggest that cellular senescence, cell cycle, and ubiquitination pathway play a major role in the early embryonic development, and the hub genes of *CCNA2* and *CCNB1* may play a crucial role in this process.

## Data Availability

The datasets presented in this study can be found in online repositories and will be made publicly available on 21.04.2025. The names of the repository/repositories and accession number(s) can be found below: https://www.ncbi.nlm.nih.gov/sra/PRJNA947755.

## Supplementary material

SUPPLEMENTARY FIGURE S1 Molecular function and cellular component associated with differentially expressed genes (DEGs) in developmental arrest embryos versus developmental normal embryos. **(A)** Molecular function of downregulated DEGs in developmental arrest embryos versus developmental normal embryos. **(B)** Cellular component associated with downregulated DEGs in developmental arrest embryos versus developmental normal embryos. **(C)** Cellular component of upregulated DEGs in developmental arrest embryos versus developmental normal embryos. **(D)** Molecular function associated with upregulated DEGs in developmental arrest embryos versus developmental normal embryos. The percentage of gene ratio is represented on the X-axis, while the Y-axis represents the GO terms. The gene count is indicated by the size of each circle. The color of circles indicates a different -log10 (*p*-value).
