# Supplementary figures and images for: High-throughput sequencing reveals hub genes for human early embryonic development arrest in vitro fertilization: a pilot study
Source: Front Physiol. 2023 Nov 8;14:1279559. doi: 10.3389/fphys.2023.1279559 (PMC10684309; doi:10.3389/fphys.2023.1279559)

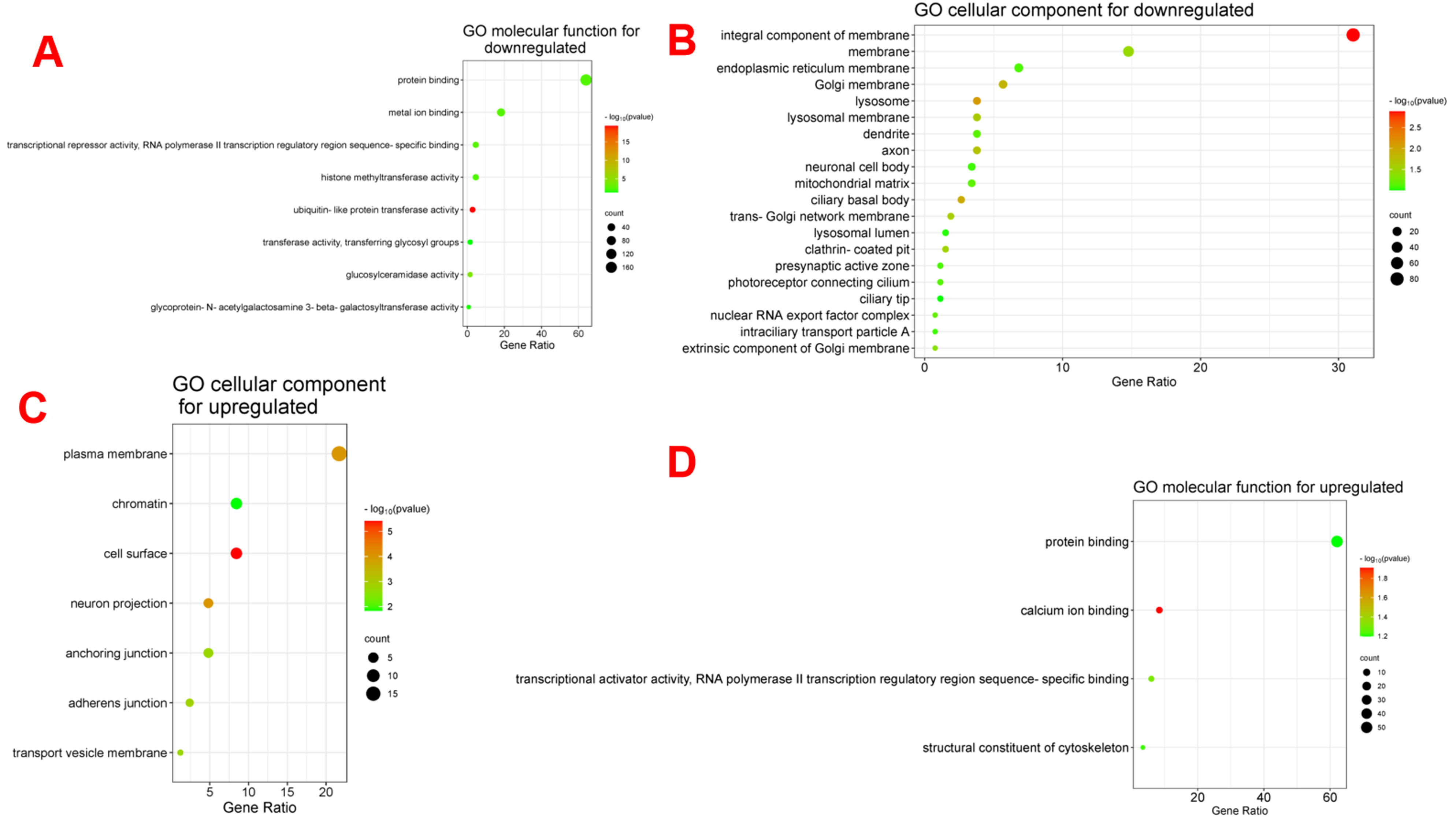

Supplement: Supplementary file 1 [file Image1.tif]
