# Supplementary material for: High-throughput sequencing reveals hub genes for human early embryonic development arrest in vitro fertilization: a pilot study
Source: Front Physiol. 2023 Nov 8;14:1279559. doi: 10.3389/fphys.2023.1279559 (PMC10684309; doi:10.3389/fphys.2023.1279559)
Supplement: Supplementary file 2 [file Table1.doc]

**Supplementary Table 1**

Clinical characteristics of subjects.

| Characteristics DNE (n =3) DAE (n =3) P value |
| --- |

Age (years) 28.37 ± 1.2135 29.70 ± 0.5763 0.4269

BMI 23.16 ± 0.8630 22.75 ± 0.3169 0.1370

| Note: Values are means ± SEM, developmental normal embryos (DNE) group (n= 3) and developmental arrest embryos (DAE) group (n= 3). |
| --- |
